# Trigonocephaly and Cranium Bifidum Occultum Treated Simultaneously Using the Split-Bone Technique and Piezosurgery

**DOI:** 10.7759/cureus.15346

**Published:** 2021-05-31

**Authors:** Leopoldo Mandic Ferreira Furtado, José Aloysio Da Costa Val Filho, José Antônio Lima Vieira, Aieska Kellen Dantas dos Santos

**Affiliations:** 1 Pediatric Neurosurgery, Vila Da Serra Hospital, Nova Lima, BRA; 2 Pediatric Neurosurgery, Vila da Serra Hospital, Nova Lima, BRA; 3 Neurosurgery, Biocor Instituto, Nova Lima, BRA

**Keywords:** craniosynostosis, cranium bifidum occultum, piezosurgery, split-bone, trigonocephaly

## Abstract

Cranium bifidum occultum (CBO) is a rare congenital disease characterized by the anomalous ossification of parietal bones, which presents with a midline bone defect with no extrusion of intracranial content. Its association with craniosynostosis has been reported only a few times to date. The aim of this case report was to describe, for the first time, the association between presumed non-syndromic trigonocephaly and CBO, as well as the treatment of both conditions using the same surgical approach. This was done by performing fronto-orbital advancement and the split-bone technique using piezosurgery, in order to achieve an autologous sample to cover the bone defect. To the best of our knowledge, this approach was proven to be safe and able to treat both diseases without a heterologous bone graft.

## Introduction

Cranium bifidum belongs to the spectrum of neural tube defects and can present with the herniation of intracranial content through a bone defect. In these cases, the condition is designated as cranium bifidum cysticum, or cranium bifidum occultum (CBO) if no extrusion of intracranial tissue is observed [1,2]. The incidence of cranium bifidum ranges from one in every 15,000 to one in every 25,000 live births [3-6] and is related to a deficiency in ossification of the parietal notch, which usually occurs at the fifth month of gestation. In addition, genetic mutations have been reported [6].

Trigonocephaly is the second most common craniosynostosis, with an estimated prevalence of one in every 5,000 live births in 2013 [7-9]. It is characterized by early fusion of the metopic suture, which presents with a restriction of frontal growing, dysmorphic orbital roofs, and skulls with a triangular shape [9]. These characteristics lead to neurodevelopmental impairment, and surgical correction is required [8]. For instance, fronto-orbital advancement (FOA) is a classical surgical technique to treat trigonocephaly and is able to expand the anterior intracranial compartment and revert the narrowing of orbits [7,8]. Almost 94% of trigonocephalic patients present in a non-syndromic way, in which only specific features of trigonocephaly with no facial dysmorphology are observed [8,9].

The association between CBO and craniosynostosis has been reported few times in the literature, and only one case report has described a patient treated for both conditions - in that case, using two separate approaches [1-3]. In addition, this combined pathophysiology was not fully understood in the past [1].

In this case report, we sought to report, for the first time, the association between trigonocephaly and CBO, as well as provide a brief literature review. Furthermore, we describe a technique using piezosurgery to obtain an autologous bone graft to cover the bone defect of CBO, using the same approach of FOA.

## Case presentation

**Figure 1:**
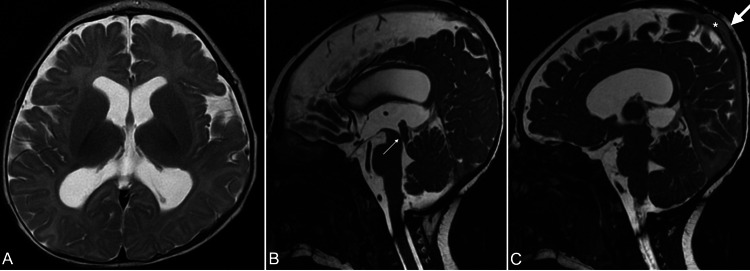
The magnetic resonance imaging of a four-month-old girl with cranium bifidum occultum The axial T2-weighted MRI demonstrates a mild dilation of lateral ventricles, with no enlargement of the third ventricle (A). The sagittal T2-weighted MRI depicts a narrow cerebral aqueduct (thin white arrow), and the parasagittal T2-weighted MRI reveals a small bone defect (thick white arrow) above the sagittal sinus (*), corresponding to cranium bifidum occultum.

A girl was born at full term and presented with ventriculomegaly and no clinical signs of hydrocephalus were observed. The delivery was uneventful. During the follow-up, the head measurement revealed a regular pattern of growing, and the transfontanelle ultrasound demonstrated no increasing of ventricle size. The anterior fontanel had regular tension. At four months old, in a further investigation with magnetic resonance imaging (MRI), a narrowing of the cerebral aqueduct was identified, as was a cystic pattern in the posterior segment of the third ventricle, leading to inferior displacement of the vein of Galen. A bone defect with 10 mm in diameter was observed superior to the sagittal sinus, with no extrusion of the intracranial content (Figure 1A-1C).

On physical exam, this defect was placed anterior to the lambda, and no skin abnormalities were observed.

**Figure 2:**
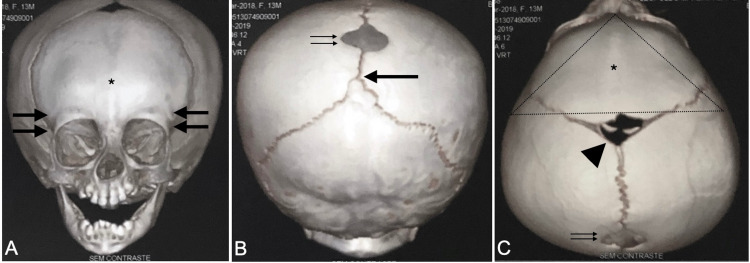
The computerized tomography scan of the skull revealing the cranium bifidum occultum associated with trigonocephaly This 3D reconstruction of a CT scan of the skull depicts trigonocephaly associated with a midline defect in the parietal bone. In the anterior view of skull, a fused metopic suture (*) and bitemporal narrowing (double thick black arrows) can be observed (A). The posterior view displays a closed posterior fontanelle (thick black arrow) and a bone defect (double thin black arrows), corresponding to cranium bifidum (B). The superior view depicts a trigonocephalic shape of the skull (dot point triangle) with an open anterior fontanelle (arrowhead) (C).

In spite of these findings, the girl did not present abnormalities in milestones or in the pattern of head growing. At seven months old, she evolved with hypoteleorbitism, which includes temporal narrowing and a trigonocephalic shape. Computed tomography (CT) was performed, and metopic fusion with frontal and orbital restriction of growth was observed, as was a midline defect occupying the parietal bones above the intersection of the sagittal and lambdoid sutures (Figures 2A-2C).

The girl had no syndromic signs, such as facial or finger abnormalities, on the physical exam. In addition, no epilepsy or irritability was observed, and the child reached all neurologic milestones. Thus, she was diagnosed with trigonocephaly and CBO.

We proposed a surgical treatment to the parents, focusing on craniosynostosis. Since the perforation of the parietal foramina in the brain carries a small risk of brain damage due to accidental head trauma, and the parents showed anxiety at this risk, we decided to treat the trigonocephaly and the bone defect at the same time surgically.

Surgical technique

The six-month-old patient was placed in a supine position, and the biparietal skin incision was fashioned with gentle curves. A technique that spares excessive blood loss was used, as previously published by our pediatric neurosurgery team [10]. At this time, the pericranium was preserved, and after a bifrontal craniotomy, the orbitectomy was performed using the Piezosurgery Mectron (Mectron Medical Technology, Genoa, Italy). A subgaleal dissection using the same biparietal incision allowed us to reach the parietal foramina posteriorly. After exposing it, the measurement was obtained using a gelfoam sponge and methylene blue. A bone sample with the exact shape of the bone defect was removed from the right frontal flap in the posterior boundary.

**Figure 3:**
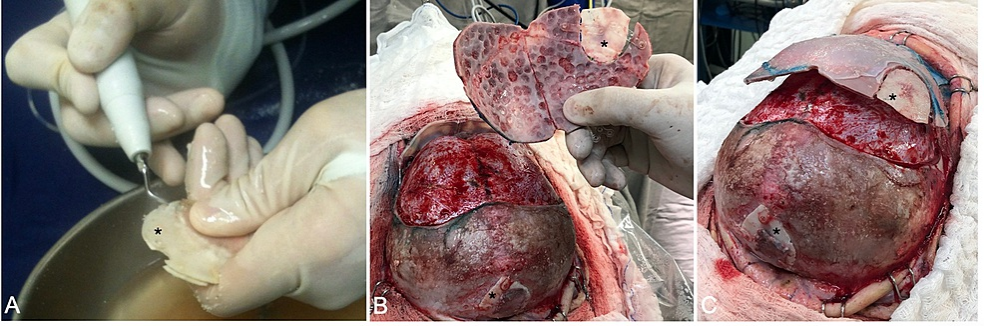
The surgical technique including fronto-orbital advancement and cranium bifidum covering The split-bone technique was used, dividing the frontal bone sample (*) using  the Piezosurgery Mectron (A). The inner surface of the frontal bone can be observed, with one partial sample fixed with two absorbable plates. Many incomplete burr holes were created in the inner surface of frontal bones to obtain more bone grafts and to cover the gaps in the frontal advancement (B). After fixation of the bandeau and frontal bone, the graft was observed covering the cranium bifidum and the frontal bone. No absorbable plates were placed in the posterior boundary of the frontal bone with the fronto-orbital technique (C).

This sample was divided using the Piezo Sonic blade, and the two resulting grafts were used to cover the cranium bifidum and the sample’s original place on the frontal bone (Figures 3A-3C).

The procedure lasted three hours, and no hemotransfusion was needed.

**Figure 4:**
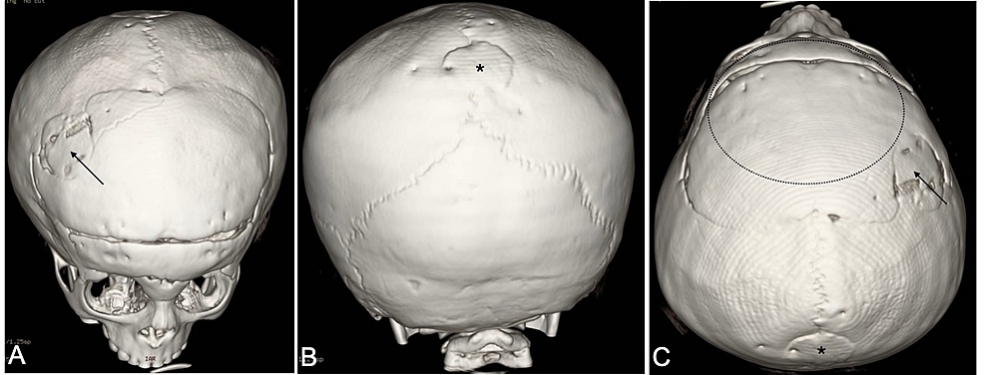
Computerized tomography of skull one year later of reconstructive surgery and treatment of trigonocephaly and cranium bifidum occultum This 3D reconstruction of a CT scan 12 months after surgery reveals the proper ossification of the autologous bone grafts in the frontal bone (thin black arrow) and cranium bifidum (*), as well as the reshaping of the skull, which acquired a round shape (dot ellipse) with the resolution of trigonocephaly.

Post-operatively, the patient developed no complications and was discharged from the hospital after five days. The patient was followed up with monthly appointments and displayed adequate skin healing and reshaping of the head. An increase in interpupillary distance and improvement of hypoteleorbitism was observed. One year after the surgery, a CT scan of the skull was performed, and we observed the proper ossification of the bone sample and reshaping of the skull (Figures 4A-4C).

## Discussion

CBO has been given other designations, such as enlarged parietal foramina (if more than 5 mm in diameter), foramina parietalia permagna, fenestrae parietals symmetricae, or Catlin mark (due to the high incidence of this bone defect in the Catlin family, as described by Goldsmith, who observed this malformation in 16 members across five generations [1-4,11,12]. Its etiology has been attributed to an abnormality in intramembranous ossification during the fifth month of gestation, and several gene mutations have been described that make this condition hereditary in nature, such as loss of function mutations in human homeobox genes MSX2 in chromosome 5 and ALX4 in chromosome 11 [3,5,11,12]. Interestingly, a deletion in chromosome 11q24 and a mutation in ALX4 are two of the mutations described in association with syndromic trigonocephaly, and an increasing incidence of this craniosynostosis has been reported over the last two decades [8,9,13]. Folic acid intake is also considered to be a risk factor that explains some of the increased incidences of trigonocephalic cases, and this factor has the potential to explain the genesis of cranium bifidum [13]. Therefore, epigenetic and environmental factors could also play a role in the development of metopic synostosis and CBO.

Furthermore, even in non-syndromic craniosynostosis cases, mutations in specific genes, such as Transcription Factor 12 (TCF12), Ephrin B1 (EFBN1), Cytochrome P450 Oxidoreductase (POR), Fibroblast Growth Factor 10 (FGF10), FRAS1 Related Extracellular Matrix 1 (FREM1), and Smad Family Member 6 (SMAD6) have been identified, specifically in trigonocephaly [13]. Nonetheless, in the present case report, no familial inheritance was observed and no mutations were identified. Moreover, the patient had no characteristic facial dysmorphisms or psychomotor retardation, as reported in almost 6% of trigonocephalic patients with specific mutations [13].

The bone defect in the current case was round and anterior to the intersection of the sagittal and lambdoid sutures, and in relation to the superior sagittal sinus, with no midline ridge. Other reports have described this defect as having a thin bone ridge in the midline [2,3,5]. In addition, the anomalous communication between the vein of Galen and the superior sagittal sinus, called persistent falcine sinus, has been reported in association with CBO [2]. Our report showed a dislodged vein of Galen due to a cystic formation. However, no communication was observed with the sagittal sinus.

Dossani et al. [1] describe a case report in which a craniofacial disorder associated with CBO was approached with posterior vault reconstruction of the CBO defect treatment, followed by a bifrontal craniotomy and orbital box osteotomies for the correction of orbital hypertelorism. They used recombinant human bone morphogenetic protein 2(rhBMP-2) along with biparietal split-bone grafts, with satisfactory results. Our case report is different, due to the association between CBO, trigonocephaly, and the use of a completely autologous graft, thanks to the ability of piezosurgery to obtain tailored frontal bone graft. No allograft was used. The argument for covering this bone defect is the potential risk of injury to the superior sagittal sinus due to trauma, even though this has been reported to be a rare event [14].

The use of piezosurgery in pediatric neurosurgery has been broadly reported in the literature, and the main advantages of this tool are the ability to provide precise cuts, minimize blood loss, and avoid the harm of underlying soft tissue, as well as for bone remodeling purposes [15-20]. Taking this into account, piezosurgery is used for the split-bone technique with bone grafts and previously has been described as a strategy to obtain a more precise bone sample to cover bone defects [19]. In the present case report, we achieved a precise bone sample with satisfactory ossification one year after the procedure.

Although no cytogenetic alterations were identified in the patient, the association of the midline defect and presumed non-syndromic trigonocephaly sheds light on the likely genetic nature of this condition. This supports the statement that patients with craniosynostosis without genetic molecular alteration may in fact have a mutation.

## Conclusions

Presumed non-syndromic trigonocephaly associated with CBO is an exceedingly rare condition. To the best of our knowledge, this case report serves as a warning to neurosurgeons, as well as maxillofacial and plastic surgeons, that non-syndromic craniosynostosis should be investigated in terms of genetic mutations, and taken into account during familiar counseling. That being said, there are other potential factors that can lead to this condition. The unique and rare case presented here illustrates possible risk factors, as well as a method to address both non-syndromic trigonocephaly and CBO in a single surgery. Furthermore, developments in FOA using piezosurgery to obtain a bone sample appear to present an available and safe option to correct this bone defect.
